# Peripheral vitamin D levels in ankylosing spondylitis: A systematic review and meta-analysis

**DOI:** 10.3389/fmed.2022.972586

**Published:** 2022-08-26

**Authors:** Maohui Diao, Jun Peng, Daidong Wang, Hongbo Wang

**Affiliations:** Department of Spine Surgery, Shenzhen Hospital of Integrated Traditional Chinese and Western Medicine, Shenzhen, China

**Keywords:** ankylosing spondylitis, meta-analysis, vitamin D, systematic review, 1, 25-dihydroxyvitamin D3

## Abstract

**Objectives:**

Previous studies showed conflicting results regarding peripheral vitamin D levels in ankylosing spondylitis (AS). We performed this systemic review and meta-analysis to explore whether vitamin D may influence AS process.

**Methods:**

Articles published until March 2022 were searched in databases as follows: PubMed, Web of Science, and Google Scholar. The present study included cross-sectional and case-control studies regarding vitamin D levels in patients with AS. Studies were excluded according to the following exclusion criteria: (1) we excluded studies which did not provide sufficient information regarding the comparison of vitamin D levels in AS patients and healthy controls (HC). Vitamin D levels in the two group studies should be reported or could be calculated in included studies; (2) meta-analysis, reviews and case reports. STATA 12.0 software was used to make a meta-analysis. Standard mean differences (SMDs) and 95% confidence intervals (CIs) were computed as effect size.

**Results:**

The present meta-analysis showed no significant difference in peripheral 1,25-dihydroxyvitamin D3 (1,25OHD) levels between AS and healthy controls (HCs) in Caucasians with a random effects model [SMD: −0.68, 95% CI (−1.90, 0.54)]. Patients with AS had lower peripheral 25-hydroxyvitamin D (25OHD) levels compared with HC with a random effects model [SMD: −0.45, 95% CI: (−0.70, −0.20)]. Patients with AS had higher peripheral C-reactive protein (CRP) and erythrocyte sedimentation rate (ESR) levels compared with HC in Caucasian population with random effects models [CRP: SMD: 1.08, 95% CI: (0.78, 1.37); ESR: SMD: 0.86, 95% CI: (0.39, 1.34)]. However, no significant difference in alkaline phosphatase (ALP), parathyroid hormone (PTH) or calcium levels were indicated between AS and HC in Caucasian with random effects models [ALP: SMD: 0.07, 95% CI: (−0.41, 0.55); PTH: SMD: −0.15, 95% CI: (−0.56, 0.26); calcium: SMD: −0.06, 95% CI: (−0.39, 0.26)].

**Conclusion:**

In conclusion, the study showed an inverse association between 25OHD and AS, which suggests that vitamin D may have a protective effect on AS. ESR and C-reactive protein (CRP) are important biomarkers for AS.

## Introduction

Ankylosing spondylitis (AS) is a chronic immune-mediated progressive rheumatic disease that mainly affects the axial spine, especially, the axial spine and sacroiliac joints (1). AS is characterized by progressive spinal ankylosis and chronic pain resulting in joint dysfunction and long-term disability (2). It was estimated that the prevalence of AS ranged from 7.4 to 23.8 per 10,000 (3). Up to now, the pathogenesis of AS has not been well-clarified. However, an increasing body of evidence suggests that AS is an inherited disease, particularly, closely genetic associated with HLA-B27 (4). Issue data reported that AS could lead to a series of complications, namely, iritis, osteoporosis, spinal compression fractures, and cardiovascular disease (5). AS leads to a life-long impact on patients and increases society burden because of the common comorbidities and chronic progressive course of work disability (6).

Vitamin D is a fat-soluble that is important in mineral and bone metabolism. The natural way to obtain vitamin D is through safe sunlight exposure and daily diet supplement (7). Vitamin D has multiple biological functions, such as stimulating calcium and phosphate absorption in the intestine, regulating parathyroid hormone (PTH) secretion (8). Vitamin D plays a role in other beneficial actions, namely, improving immune function, reducing the risk of cancer death, and preventing cardiovascular complications (9, 10).

There are increasing data linking vitamin D and the immune system. Vitamin D deficiency has been described as a risk factor in the progression of several autoimmune diseases such as multiple sclerosis and Crohn's disease, generally attributed to the potent immunomodulatory effects of 1,25-dihydroxyvitamin D3 (1,25OHD) (11, 12). Klingberg et al. reported that serum 25-hydroxyvitamin D (25OHD) in patients with AS was not different from healthy controls (HCs), and not associated with disease activity (13), whereas Fotoh et al. reported that there was a significant difference in serum 25OHD between patients with HC and AS (14). Because of these conflicting results, we performed this systemic review and meta-analysis to explore whether vitamin D may influence AS process.

## Methods

The present study was conducted drawing on the Preferred Reporting Items for Systematic Reviews and Meta-Analyses (PRISMA) statement (15). The present study is a meta-analysis, ethical approval was not applicable.

### Search strategy and selection criteria

Articles published until March 2022 were searched in databases as follows: PubMed, Web of Science, and Google Scholar. Search terms (“ankylosing spondylitis” OR “AS”) AND (“vitamin D” OR “25OHD” OR “1,25OHD”) were used.

The present study included cross-sectional and case-control studies regarding vitamin D levels in patients with AS. Studies were excluded according to the following exclusion criteria: (1) we excluded studies which did not provide sufficient information regarding the comparison of vitamin D levels in patients with AS and HC. Vitamin D levels in the two group studies should be reported or could be calculated in included studies; (2) meta-analysis, reviews, and case reports.

### Data extraction and meta-analysis

In total, two investigators extracted data from finally included studies independently. Extracted data showed as follows: author, publication year, study location, sample size, gender, mean age, and results. STATA 12.0 software was used to make meta-analysis. Standard mean differences (SMDs) and 95% confidence intervals (CIs) were computed as effect size. *Q*-test and *I*^2^ were used to assess heterogeneity among studies. In addition, meta-regression analysis was conducted to detect source of the heterogeneity. With high heterogeneity (*p*-value of *Q* test ≤ 0.05 and *I*^2^ ≥ 50%), random effects models were used to compute results; with low heterogeneity (*p*-value of *Q* test > 0.05 and *I*^2^ <50%), fixed effects models were used. Subgroup analyses (for different ethnicities) were used to investigate the effect of ethnicities on results. In addition, the stability of the meta-analysis was evaluated with sensitivity analysis. Moreover, the Begg's test, Egger's test, and funnel plots were made to evaluate publication bias.

## Results

### Selection results

Selection procedures were illustrated in Supplementary Figure 1. Supplementary Table 1 showed study characteristics of 15 finally included studies (13, 14, 16–28). These studies included 2,703 patients with AS and 6,145 HC.

### Meta-analysis results

**Figure 1:**
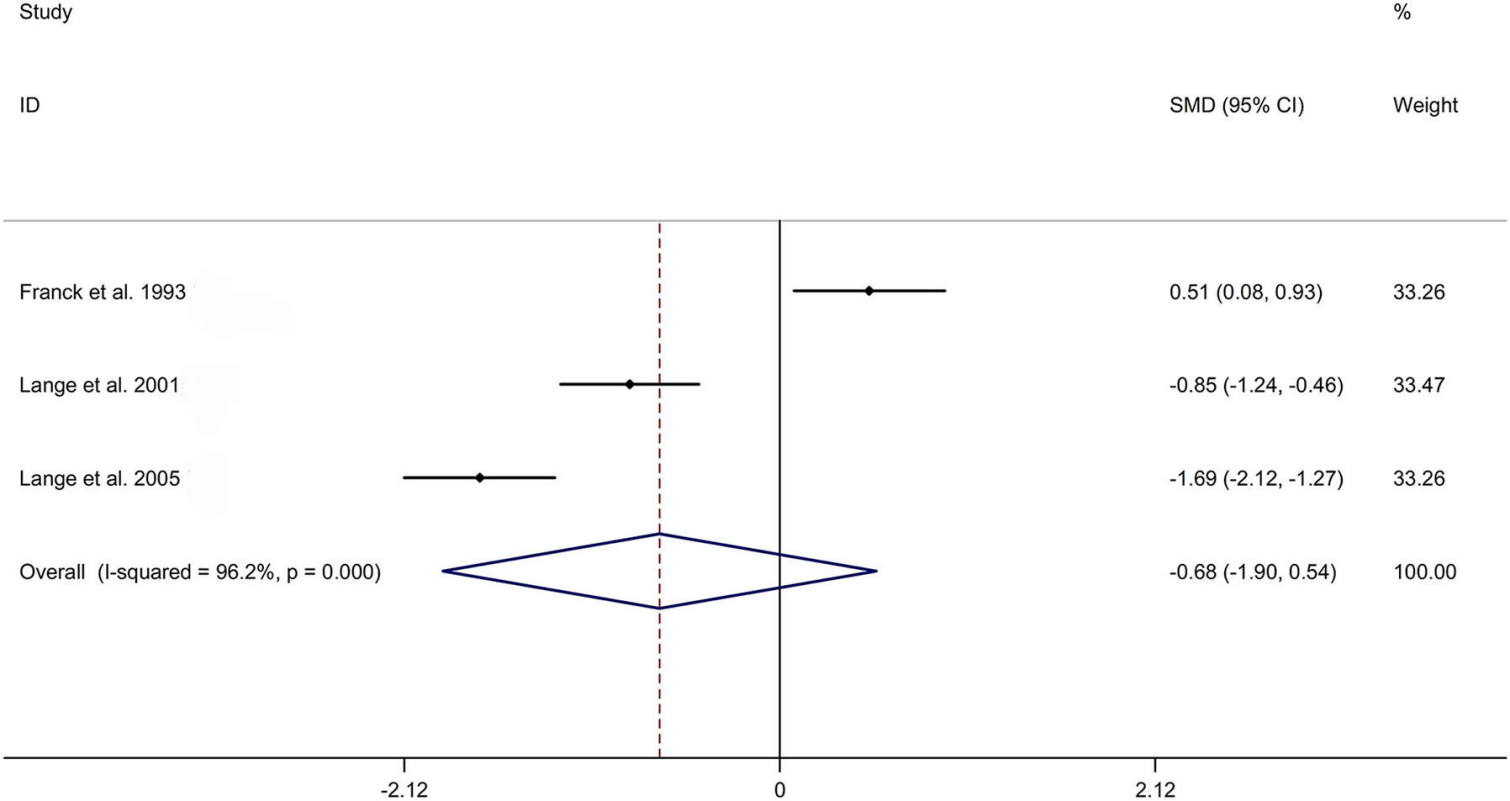
Forest plot regarding comparison in peripheral 1,25OHD levels between AS and HC. AS, ankylosing spondylitis; HC, healthy controls; 1,25OHD, 1,25-dihydroxyvitamin D3.

**Figure 2:**
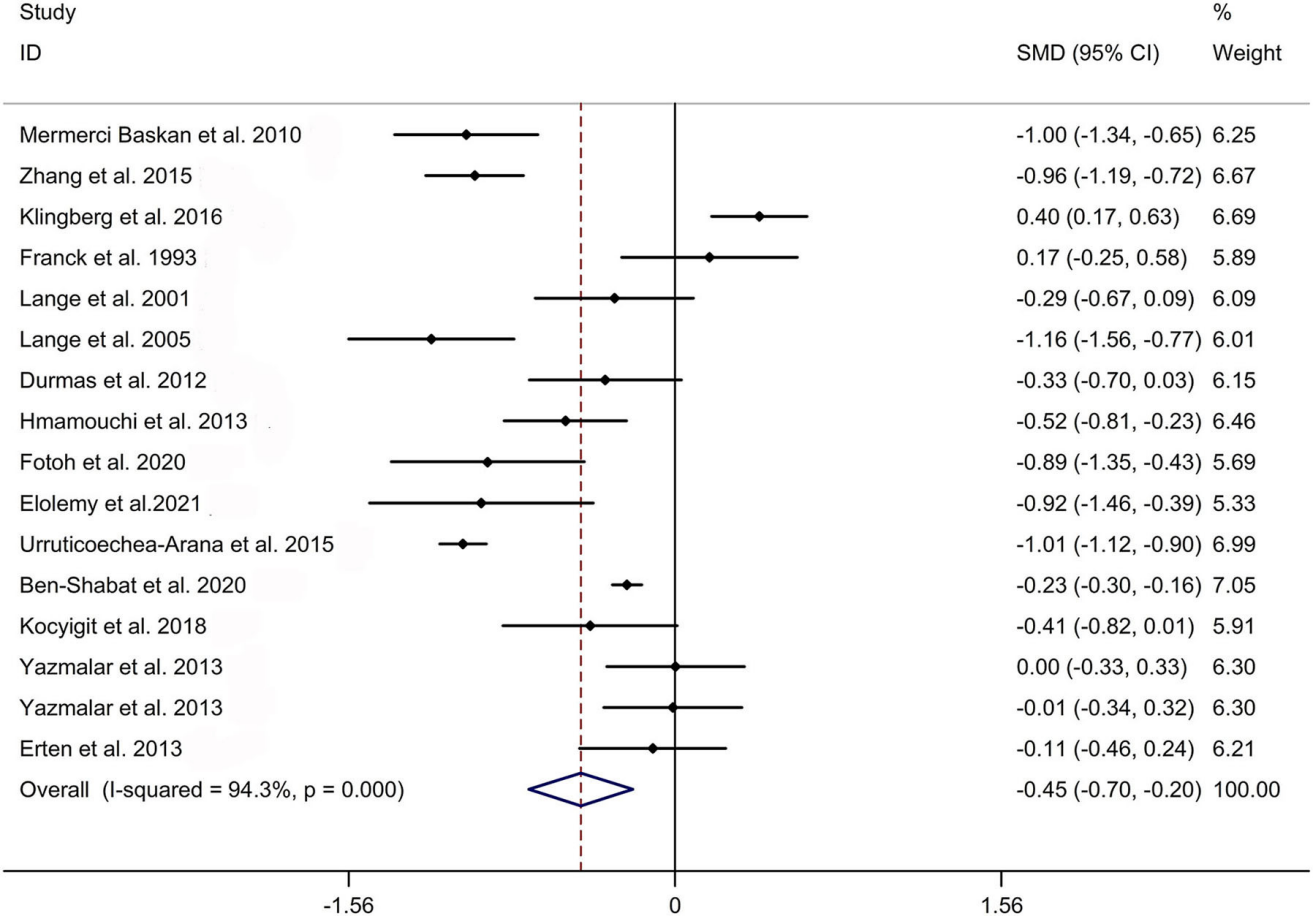
Forest plot regarding comparison in peripheral 25OHD levels between AS and HC. AS, ankylosing spondylitis; HC, healthy controls; 25OHD, 25-hydroxyvitamin D.

**Figure 3:**
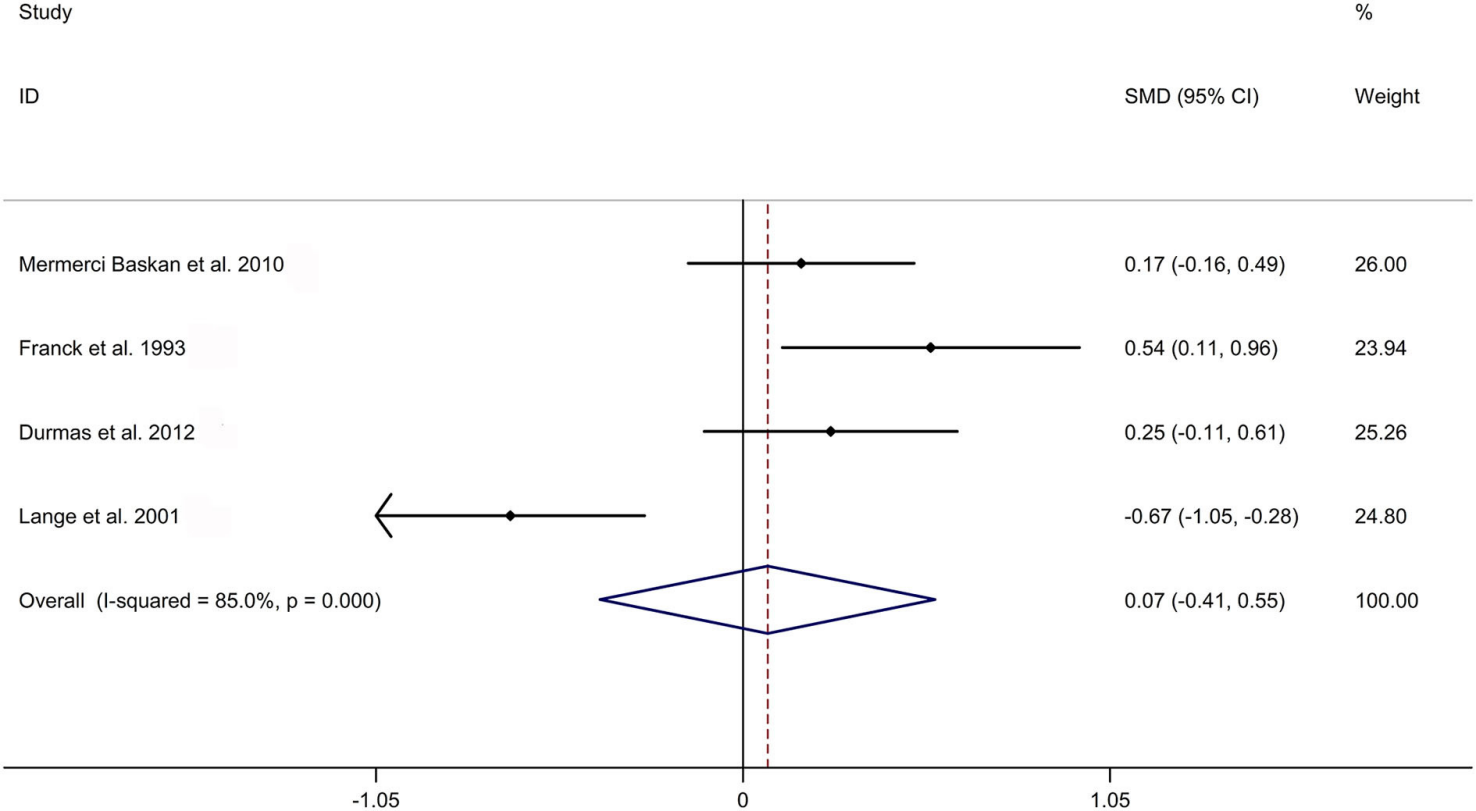
Forest plot regarding comparison in peripheral ALP levels between AS and HC. ALP, alkaline phosphatase; AS, ankylosing spondylitis; HC, healthy controls.

**Figure 4:**
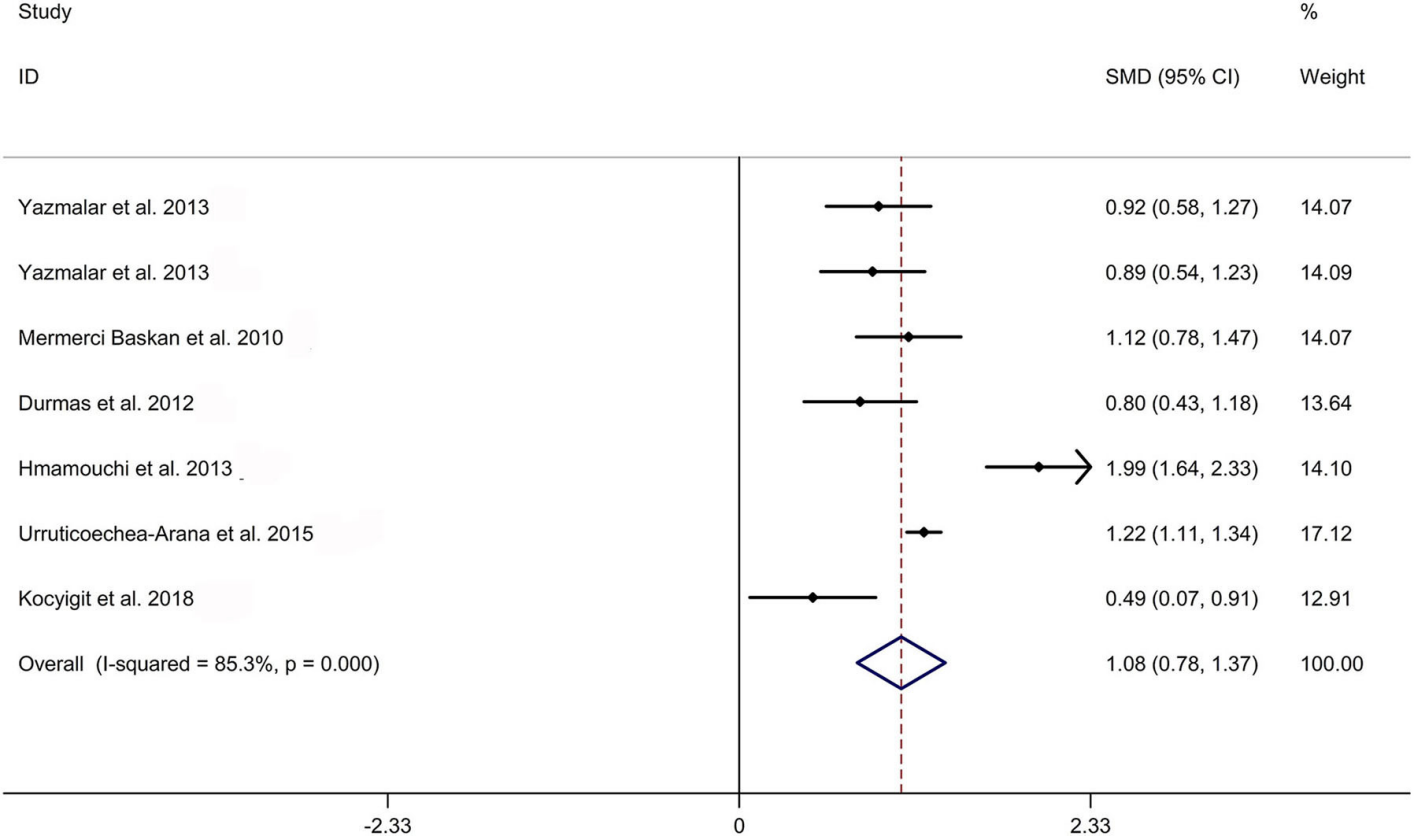
Forest plot regarding comparison in peripheral CRP levels between AS and HC. AS, ankylosing spondylitis; CRP, C-reactive protein; HC, healthy controls.

**Figure 5:**
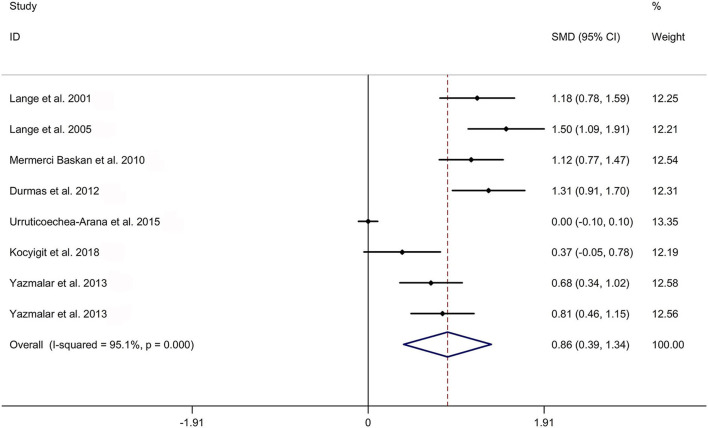
Forest plot regarding comparison in peripheral ESR levels between AS and HC. AS, ankylosing spondylitis; ESR, erythrocyte sedimentation rate; HC, healthy controls.

**Figure 6:**
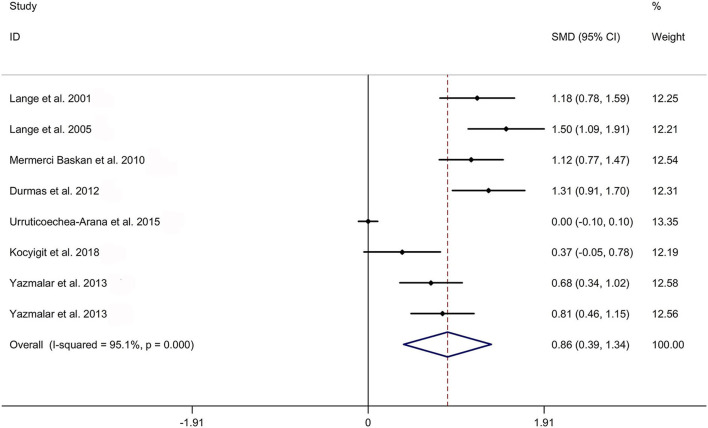
Forest plot regarding comparison in peripheral PTH levels between AS and HC. AS, ankylosing spondylitis; HC, healthy controls; PTH, parathyroid hormone.

**Figure 7:**
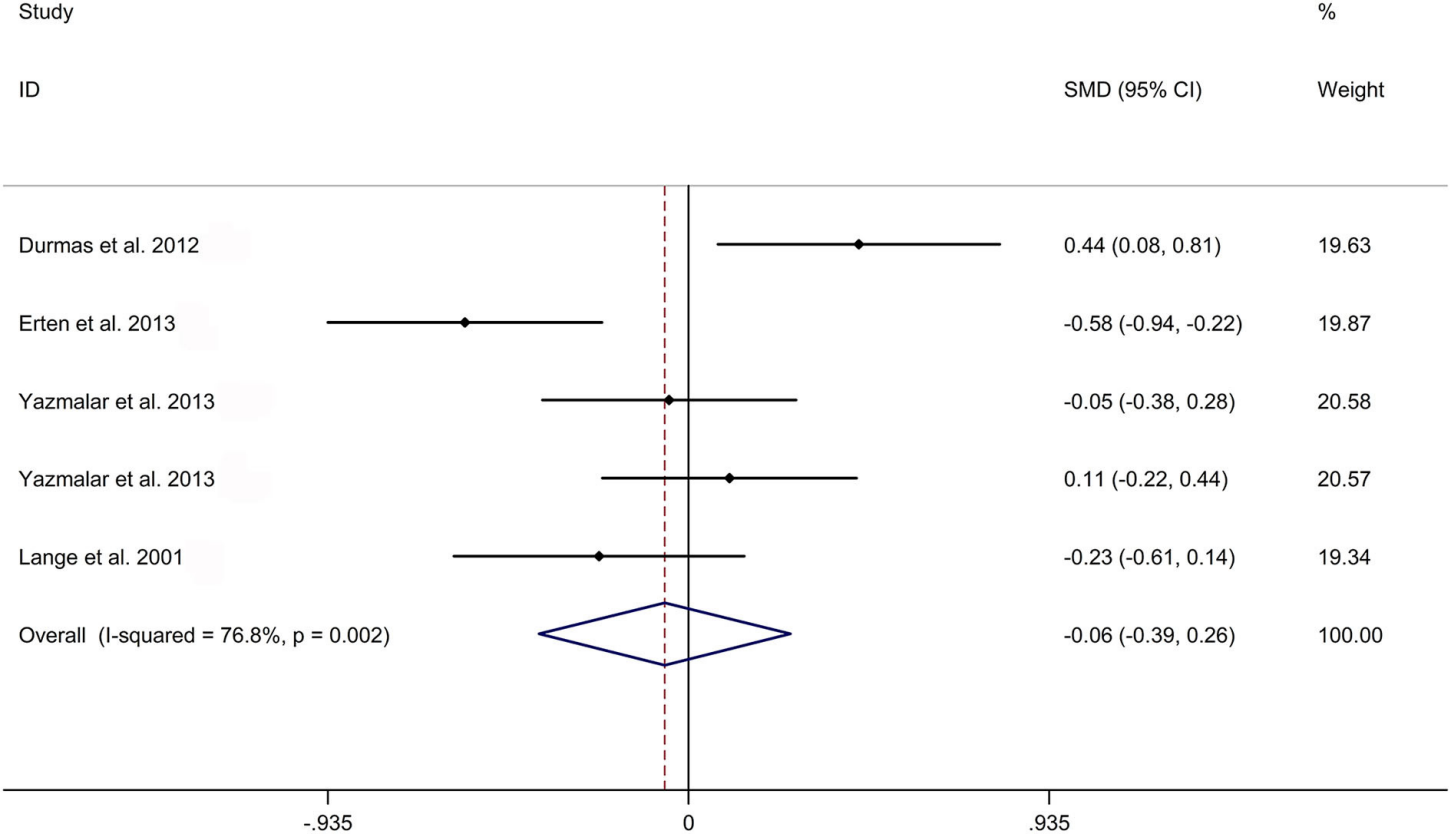
Forest plot regarding comparison in peripheral calcium levels between AS and HC. AS, ankylosing spondylitis; HC, healthy controls.

The present meta-analysis showed no significant difference in peripheral 1,25OHD levels between AS and HC in Caucasian with a random effects model [SMD: −0.68, 95% CI (−1.90, 0.54); *p*-value of *Q* test <0.001, *I*^2^ = 96.2%; Figure 1]. Patients with AS had lower peripheral 25OHD levels compared with HC with a random effects model [SMD: −0.45, 95% CI: (−0.70, −0.20); *p*-value of *Q* test <0.001, *I*^2^ = 94.3%; Figure 2]. Subgroup studies showed no significant difference in peripheral 25OHD levels between patients with AS and HC in the Caucasian population [SMD: −0.41, 95% CI: (−0.67, −0.16); Supplementary Figure 2]. No significant difference in alkaline phosphatase (ALP) levels were indicated between AS and HC in Caucasian with a random effects model [SMD: 0.07, 95% CI: (−0.41, 0.55); *p*-value of *Q* test <0.001, *I*^2^ = 85.0%; Figure 3]. Patients with AS had higher peripheral C-reactive protein (CRP) levels compared with HC in the Caucasian population with a random effects model [SMD: 1.08, 95% CI: (0.78, 1.37); *p*-value of *Q* test <0.001, *I*^2^ = 85.3%; Figure 4]. In addition, patients with AS had higher peripheral erythrocyte sedimentation rate (ESR) levels compared with the HC in the Caucasian with random effects models [SMDs: 0.86, 95%CI: (0.39, 1.34); *p*-value of *Q* test <0.001, *I*^2^ = 95.1%; Figure 5], whereas no significant difference in PTH or peripheral calcium levels was showed between AS and HC in the Caucasian with a random effects model [PTH: SMD: −0.15, 95% CI: (−0.56, 0.26); *p*-value of *Q* test <0.001, *I*^2^ = 91.7%; Figure 6; calcium: SMD: −0.06, 95% CI: (−0.39, 0.26); *p*-value of *Q* test = 0.002, *I*^2^ = 76.8%; Figure 7].

### Meta-regression results

Meta-regression analysis showed that ages and gender were not responsible for heterogeneity across studies regarding comparison of peripheral 25OHD, ALP, CRP, ESR, PTH, and calcium levels between patients with AS and HC (Supplementary Table 2).

### Results of sensitivity analysis and publication bias

Sensitivity analyses showed no changes in the direction of effect when any one study was excluded for all meta-analyses (Supplementary Figure 3). Supplementary Table 3 and Supplementary Figure 4 showed results of publication bias. The Begg's test, Egger's tests, and funnel plots showed no significant risks of publication bias for meta-analyses regarding comparison of peripheral 1,25OHD, 25OHD, ALP, CRP, PTH, and calcium between patients with AS and HC (Supplementary Table 3 and Supplementary Figures 4A–G), whereas the Begg's test, Egger's tests, and funnel plots showed significant risks of publication bias for meta-analyses regarding comparison of ESR between patients with AS and HC (Supplementary Table 3 and Supplementary Figure 4E)

## Discussion

In this systemic review and meta-analysis, we found that there was a significant difference between HC and patients with AS in the level of serum 25OHD, whereas no difference in 1,25OHD. This result was consistent with the article published by Cai et al. in 2015 which reported that the SMD of 25OHD between patients with AS and controls was −0.66 (−1.08, −0.24), and 1,25OHD was −0.72 (−1.79, 0.35) (29). The conversion of vitamin D into the active hormone 1,25OHD needs two hydroxylation steps. The concentration of 1,25OHD, which is in the order of pg/ml, is much lower than 25OHD (order of ng/ml) in the blood, thus 25OHD is more easily to be detected than 1,25OHD (30). Moreover, the conversion rate of 1,25OHD is regulated by many paracrine and autocrine factors including PTH concentrations (31). In addition, 1,25OHD is with a short half-life between 4 and 6 h. Therefore, 1,25OHD is not associated with patients with AS, which may be because of the instability of 1,25OHD in serum. This result needs more convincing evidence to be confirmed.

In total, 1,25OHD and vitamin D are recognized as important immune system regulators that is widely accepted and the vitamin D receptor (VDR) has been found in the immune system (32). Murdaca et al. reported the important role of vitamin D and the gut microbiome in the efficiency of the immune response (33). Interactions between vitamin D, gut microbiome, and the immune system may happen at several levels and can include both the innate and the adaptive immune systems (33). Immune system and the microbiome are interconnected, and vitamin D plays a critical role in this dynamic (33). In total, 1,25OHD works mainly by means of VDR (34). VDR is a transcription factor and nuclear hormone receptor expressed in some tissues, namely, the intestines, liver, adipose tissue, and most immune cells. VDR modulates procedures of metabolic and immune system (35). Because VDR expresses in most immune cells (including CD4+ and CD8+ T cells, B cells, antigen-presenting cells (APCs), and neutrophils), it plays a critical role in the modulation of the immune response (36). In addition, VDR is also highly expressed in the small intestine and colon, where it plays a critical role in immunity, host-microbial interactions, and susceptibility to pathogenic infection. VDR expressed in the intestines is critical for maintaining a healthy microbiome (37). Changes in vitamin D/VDR signaling are related to microbiome dysbiosis, which in turn has been associated with both intestinal inflammatory processes and extra-intestinal conditions such as AS. A previous study supported that vitamin D and 1,25OHD can effectively treat animal of T-cells-mediated diseases such as multiple sclerosis (38). A low level of 25OHD was associated with increasing risk of multiple sclerosis among Caucasians (39). In addition, several recently published articles provided novel evidence to support that vitamin D associated gene polymorphisms were in connection with AS. Zhang et al. showed that in Han Chinese the haplotypes (TG) of rs11168266-rs11168267 in the VDR gene was associated with AS susceptibility (40). A case-control study indicated that haplotypes of rs1544410 and rs731236 in the VDR gene significantly conferred the risk of AS (41). Furthermore, serum VDR levels have been linked to disease activity and clinical parameters among patients with AS, which may be a potential marker of AS (42). Jung et al. reported that vitamin D-binding protein gene polymorphisms were related to the risk of peripheral arthritis and uveitis among patients with AS (43).

Previous studies have concluded that there is a tightly regulated feedback cycle between vitamin D and PTH secretion (44). PTH stimulates the conversion of 25OHD to 1,25OHD in the kidney, and enhances renal calcium reabsorption (45). Thus, it is considered that serum PTH levels were inversely associated with 25OHD. In our study, no significant difference in PTH levels was showed between AS and HC in Caucasian (SMD: −0.15, 95% CI: −0.56–0.26). ESR and CRP are non-specific indicators for systemic inflammation. Our research showed that both CRP and ESR were higher in patients with AS than in controls (CRP: SMD = 1.08, 95% CI: 0.78, 1.37; ESR: SMD = 0.86, 95% CI: 0.39, 1.34).

We discussed the associations between activity of AS and several critical factors and found there were significant differences in 25OHD, ESR, and CRP between patients with AS and HC. However, there were some limitations in this meta-analysis. At first, most of included studies were based on Caucasians, and only one on yellow race. Second, because of the limitation of data resources, we could not evaluate the impact of several factors (such as diet and sunshine duration in the area where the sample population is located, and the season of blood sampling) on the association between AS and vitamin D.

In conclusion, the study showed an inverse association between 25OHD and AS, which suggests that vitamin D may have protective effect on AS. However, whether vitamin D supplement decreases the risk of AS needs further research. More well-designed studies are needed to confirm the relationship between vitamin D and AS.

## Data availability statement

The original contributions presented in the study are included in the article/Supplementary material, further inquiries can be directed to the corresponding author.

## Author contributions

MD planned the study, made data collection, and manuscript writing. JP made study search, data collection, and data analysis. DW made study search and data analysis. HW submitted the study. All authors contributed to the article and approved the submitted version.

## Conflict of interest

The authors declare that the research was conducted in the absence of any commercial or financial relationships that could be construed as a potential conflict of interest.

## Publisher's note

All claims expressed in this article are solely those of the authors and do not necessarily represent those of their affiliated organizations, or those of the publisher, the editors and the reviewers. Any product that may be evaluated in this article, or claim that may be made by its manufacturer, is not guaranteed or endorsed by the publisher.
