# Supplementary material for: Peripheral vitamin D levels in ankylosing spondylitis: A systematic review and meta-analysis
Source: Front Med (Lausanne). 2022 Aug 26;9:972586. doi: 10.3389/fmed.2022.972586 (PMC9458854; doi:10.3389/fmed.2022.972586)
Supplement: Supplementary file 1 [file Table_1.DOCX]

Supplementary table 1. Study characteristics of finally included studies.

| Study | Country | Sample size (AS/control) | Gender (male%) | Age (mean) | Results | | | |
| --- | --- | --- | --- | --- | --- | --- | --- | --- |
|  |  |  |  |  | Measurement | | AS | Control |
| Franck et al. 1993 ^[1]^ | Germany | 38/52 | 60% | 39.8 | 25OHD (ng/ml) | | 21.6 (13.5) | 20.1 (3.2) |
|  |  |  |  |  | 1,25-D (pg/ml) | | 64.0 (34.5) | 52.4 (6.7) |
|  |  |  |  |  | PTH (mE/ml) | | 3.1 (0.7) | 2.7 (0.6) |
|  |  |  |  |  | ALP (U/l) | | 135 (44) | 114 (35) |
| Lange et al. 2001 ^[2]^ | Germany | 70/45 | NR | NR | 25OHD (ng/ml) | | 22 (1) | 24 (11) |
|  |  |  |  |  | 1,25-D (pg/ml) | | 31 (13) | 42 (13) |
|  |  |  |  |  | PTH (pg/ml) | | 24 (15) | 37 (17) |
|  |  |  |  |  | ALP (μg/l) | | 11 (3) | 13 (3) |
|  |  |  |  |  | ESR (mm/h) | | 23 (17) | 7 (4) |
|  |  |  |  |  | serum calcium (mmol/l) | | 2.29 (0.09) | 2.31 (0.08) |
| Lange et al. 2005 ^[3]^ | Germany | 58/58 | 65.5% | 38.4 | 25OHD (ng/ml) | | 19.1 (9.8) | 29 (7) |
|  |  |  |  |  | 1,25-D (pg/ml) | | 27.2 (8.6) | 43 (10) |
|  |  |  |  |  | PTH (pg/ml) | | 16.6 (5.5) | 37 (17) |
|  |  |  |  |  | ESR (mm/h) | | 20.3 (11.9) | 7 (4) |
| Mermerci Baskan et al. 2010 ^[4]^ | Turkey | 100/58 | 76.6% | 39.2 | 25OHD (mmol/l) | | 21.70 (12.17) | 32.70 (8.77) |
|  |  |  |  |  | PTH (pmol/l) | | 3.95 (1.90) | 3.65 (1.27) |
|  |  |  |  |  | ESR (mm/h) | | 26.31 (20.54) | 7.53 (6.09) |
|  |  |  |  |  | ALP (IU/l) | | 24.83 (12.65) | 22.96 (8.20) |
|  |  |  |  |  | CRP (mg/l) | | 21.43 (19.90) | 3.57 (1.73) |
| Durmas et al. 2012 ^[5]^ | Turkey | 99/42 | 83.0% | 36.6 | 25OHD (ng/ml) | | 26.8 (11.7) | 31.1 (15.5) |
|  |  |  |  |  | PTH (pg/ml) | | 34.5 (18.0) | 30.1 (13.5) |
|  |  |  |  |  | ALP (U/l) | | 88.2 (44.6) | 78.1 (27.1) |
|  |  |  |  |  | ESR (mm/h) | | 39.1 (28.5) | 7.5 (6.3) |
|  |  |  |  |  | CRP (mg/l) | | 21.3 (25.5) | 4.1 (2.0) |
|  |  |  |  |  | serum calcium (mg/dl) | | 9.75 (0.5) | 9.5 (0.7) |
| Yazmalar et al. 2013 ^[6]^ | Turkey | 72/70 | 69.7% | 36.2 | 25OHD (ng/ml) | summer | 30.79 (22.86) | 30.73 (18.53) |
|  |  |  |  |  |  | winter | 29.57 (30.47) | 29.82 (19.19) |
|  |  |  |  |  | PTH (pg/ml) | summer | 44.32 (19.48) | 50.78 (17.21) |
|  |  |  |  |  |  | winter | 42.97 (22.60) | 48.98 (21.43) |
|  |  |  |  |  | ESR (mm/h) | summer | 15.53 (14.45) | 7.82 (6.62) |
|  |  |  |  |  |  | winter | 16.66 (13.66) | 7.69 (7.65) |
|  |  |  |  |  | CRP (IU/ml) | summer | 12.93 (14.41) | 3.42 (1.06) |
|  |  |  |  |  |  | winter | 15.35 (19.13) | 3.27 (0.59) |
|  |  |  |  |  | serum calcium (mg/dl) | summer | 9.43 (0.38) | 9.45 (0.42) |
|  |  |  |  |  |  | winter | 9.35 (0.38) | 9.31 (0.37) |
| Erten et al. 2013 ^[7]^ | Turkey | 48/92 | 72.9% | 38.1 | 25OHD (μg/l) * | | 18 (8-38) | 20 (4-92.3) |
|  |  |  |  |  | PTH (pg/ml) * | | 75 (30-145) | 60 (13.7-172) |
|  |  |  |  |  | serum calcium (mg/dl) | | 8.9 (0.2) | 9.1 (0.4) |
| Hmamouchi et al. 2013 ^[8]^ | Morocco | 70/140 | NR | 41.3 | 25OHD (ng/ml) | | 17.5 (9.7) | 21.9 (7.7) |
|  |  |  |  |  | PTH (pg/ml) | | 64.5 (47.6) | 56.9 (24.3) |
|  |  |  |  |  | CRP (mg/l) | | 24.1 (19.6) | 1.6 (1.2) |
| Urruticoechea-Arana et al. 2015 ^[9]^ | Spain | 738/677 | 55.0% | 50.9 | 25OHD (ng/ml) # | | 20.9 (13.1-29.0) | 24.8 (18.4-32.6) |
|  |  |  |  |  | ESR (mm/h) # | | 10.0 (6.0-21.0) | 10.0 (5.0-18) |
|  |  |  |  |  | CRP (mg/l) # | | 3.6 (1.6-8.9) | 1.9 (1.3-3.3) |
| Zhang et al. 2015 ^[10]^ | China | 150/168 | 80.2% | 30.6 | 25OHD (mmol/l) | | 57.92 (24.42) | 91.24 (42.02) |
| Klingberg et al. 2016 ^[11]^ | Sweden | 203/120 | 58.8% | 45.3 | 25OHD (mmol/l) # | | 48.0 (34.0-64.0) | 45.0 (32.0-59.8) |
| Kocyigit et al. 2018 ^[12]^ | Turkey | 68/34 | 72.5% | 40.7 | 25OHD (ng/ml) * | | 14.58 (1.02-50) | 20.20 (4.02-68) |
|  |  |  |  |  | ESR (mm/h) * | | 9 (1-47) | 5 (1-35) |
|  |  |  |  |  | CRP (mg/l) * | | 5.88 (0.09-45.30) | 1.19 (0.03-11.4) |
| Ben-Shabat et al. 2020 ^[13]^ | Israel | 919/4519 | 77.2% | 52.1 | 25OHD (ng/ml) # | | 20.6 (14-26) | 21.3 (15-27) |
| Fotoh et al. 2020 ^[14]^ | Egypt | 40/40 | 77.5% | 47.2 | 25OHD (ng/ml) | | 16.9 (4.0) | 20.3 (3.6) |
| [Elolemy](https://pubmed.ncbi.nlm.nih.gov/?show_snippets=off&term=Elolemy+G&cauthor_id=33687883) et al.2021 ^[15]^ | Egypt | 30/30 | NR | NR | 25OHD (ng/ml) | | 27.73 (14.27) | 38.46 (8.11) |

Abbreviations: AS: ankylosing spondylitis; ALP, Alkaline phosphatase; PTH, parathyroid hormone; ESR, erythrocyte sedimentation rate; CRP: C-reactive protein; *: median (min-max); #: median (p25-p75).

Supplementary references

[1] FRANCK H, KECK E. Serum osteocalcin and vitamin D metabolites in patients with ankylosing spondylitis [J]. Annals of the rheumatic diseases, 1993, 52(5): 343-6.

[2] LANGE U, JUNG O, TEICHMANN J, et al. Relationship between disease activity and serum levels of vitamin D metabolites and parathyroid hormone in ankylosing spondylitis [J]. Osteoporosis international : a journal established as result of cooperation between the European Foundation for Osteoporosis and the National Osteoporosis Foundation of the USA, 2001, 12(12): 1031-5.

[3] LANGE U, TEICHMANN J, STRUNK J, et al. Association of 1.25 vitamin D3 deficiency, disease activity and low bone mass in ankylosing spondylitis [J]. Osteoporosis international : a journal established as result of cooperation between the European Foundation for Osteoporosis and the National Osteoporosis Foundation of the USA, 2005, 16(12): 1999-2004.

[4] MERMERCI BAŞKAN B, PEKIN DOĞAN Y, SIVAS F, et al. The relation between osteoporosis and vitamin D levels and disease activity in ankylosing spondylitis [J]. Rheumatology international, 2010, 30(3): 375-81.

[5] DURMUS B, ALTAY Z, BAYSAL O, et al. Does vitamin D affect disease severity in patients with ankylosing spondylitis? [J]. 中华医学杂志(英文版), 2012, 125(014): 2511-5.

[6] YAZMALAR L, EDIZ L, ALPAYCI M, et al. Seasonal disease activity and serum vitamin D levels in rheumatoid arthritis, ankylosing spondylitis and osteoarthritis [J]. African health sciences, 2013, 13(1): 47-55.

[7] ERTEN S, KUCUKSAHIN O, SAHIN A, et al. Decreased plasma vitamin D levels in patients with undifferentiated spondyloarthritis and ankylosing spondylitis [J]. Internal medicine (Tokyo, Japan), 2013, 52(3): 339-44.

[8] IHSANE, HMAMOUCHI, FADOUA, et al. The relation between disease activity, vitamin D levels and bone mineral density in men patients with ankylosing spondylitis [J]. Rheumatology Reports, 2013,

[9] URRUTICOECHEA-ARANA A, MARTíN-MARTíNEZ M A, CASTAñEDA S, et al. Vitamin D deficiency in chronic inflammatory rheumatic diseases: results of the cardiovascular in rheumatology [CARMA] study [J]. Arthritis research & therapy, 2015, 17(1): 211.

[10] ZHANG P, LI Q, WEI Q, et al. Serum Vitamin D and Pyridinoline Cross-Linked Carboxyterminal Telopeptide of Type I Collagen in Patients with Ankylosing Spondylitis [J]. BioMed research international, 2015, 2015(543806.

[11] KLINGBERG E, OLERöD G, HAMMARSTEN O, et al. The vitamin D status in ankylosing spondylitis in relation to intestinal inflammation, disease activity, and bone health: a cross-sectional study [J]. Osteoporosis international : a journal established as result of cooperation between the European Foundation for Osteoporosis and the National Osteoporosis Foundation of the USA, 2016, 27(6): 2027-33.

[12] KOCYIGIT B F, AKYOL A. Vitamin D levels in patients with ankylosing spondylitis: Is it related to disease activity? [J]. Pakistan journal of medical sciences, 2018, 34(5): 1209-14.

[13] BEN-SHABAT N, WATAD A, SHABAT A, et al. Low Vitamin D Levels Predict Mortality in Ankylosing Spondylitis Patients: A Nationwide Population-Based Cohort Study [J]. Nutrients, 2020, 12(5):

[14] FOTOH D S, SERAG D M, BADR I T, et al. Prevalence of Subclinical Carotid Atherosclerosis and Vitamin D Deficiency in Egyptian Ankylosing Spondylitis Patients [J]. Archives of rheumatology, 2020, 35(3): 335-42.

[15] ELOLEMY G, HASSAN W, NASR M, et al. Hypovitaminosis D in Patients with Ankylosing Spondylitis: Frequency and Consequences [J]. Current rheumatology reviews, 2021,
