# Supplementary material for: Peripheral vitamin D levels in ankylosing spondylitis: A systematic review and meta-analysis
Source: Front Med (Lausanne). 2022 Aug 26;9:972586. doi: 10.3389/fmed.2022.972586 (PMC9458854; doi:10.3389/fmed.2022.972586)
Supplement: Supplementary file 2 [file Table_2.DOCX]

Supplementary table 2. Meta-regression results.

| Indicator | Meta regression for age | Meta regression for gender |
| --- | --- | --- |
| 25OHD | 0.462 | 0.772 |
| ALP | 0.448 | 0.746 |
| CRP | 0.736 | 0.741 |
| ESR | 0.737 | 0.327 |
| PTH | 0.532 | 0.519 |
| Calcium | 0.238 | 0.183 |
