# Supplementary material for: Peripheral vitamin D levels in ankylosing spondylitis: A systematic review and meta-analysis
Source: Front Med (Lausanne). 2022 Aug 26;9:972586. doi: 10.3389/fmed.2022.972586 (PMC9458854; doi:10.3389/fmed.2022.972586)
Supplement: Supplementary file 3 [file Table_3.DOCX]

Supplementary table 3. Results of publication bias

| Indicator | Begg’s Test | Egger’s test |
| --- | --- | --- |
| 1,25OHD | 1.000 | 0.916 |
| 25OHD | 0.499 | 0.834 |
| ALP | 0.734 | 0.959 |
| CRP | 0.072 | 0.489 |
| ESR | 0.174 | 0.001 |
| PTH | 0.602 | 0.325 |
| Calcium | 1.000 | 0.836 |
