# Supplementary figures and images for: Peripheral vitamin D levels in ankylosing spondylitis: A systematic review and meta-analysis
Source: Front Med (Lausanne). 2022 Aug 26;9:972586. doi: 10.3389/fmed.2022.972586 (PMC9458854; doi:10.3389/fmed.2022.972586)

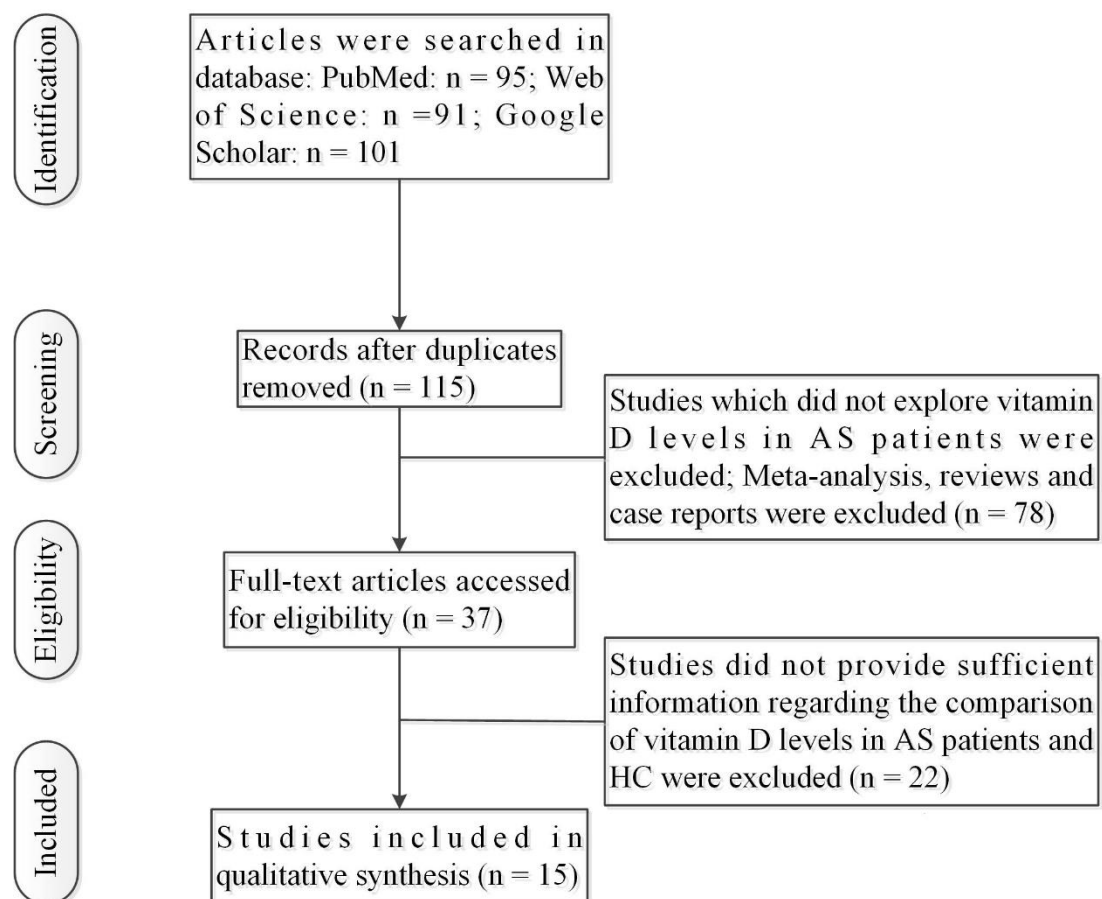

Supplementary figure 1. Selection procedures.

Supplement: Supplementary file 4 [file Image_1.pdf]

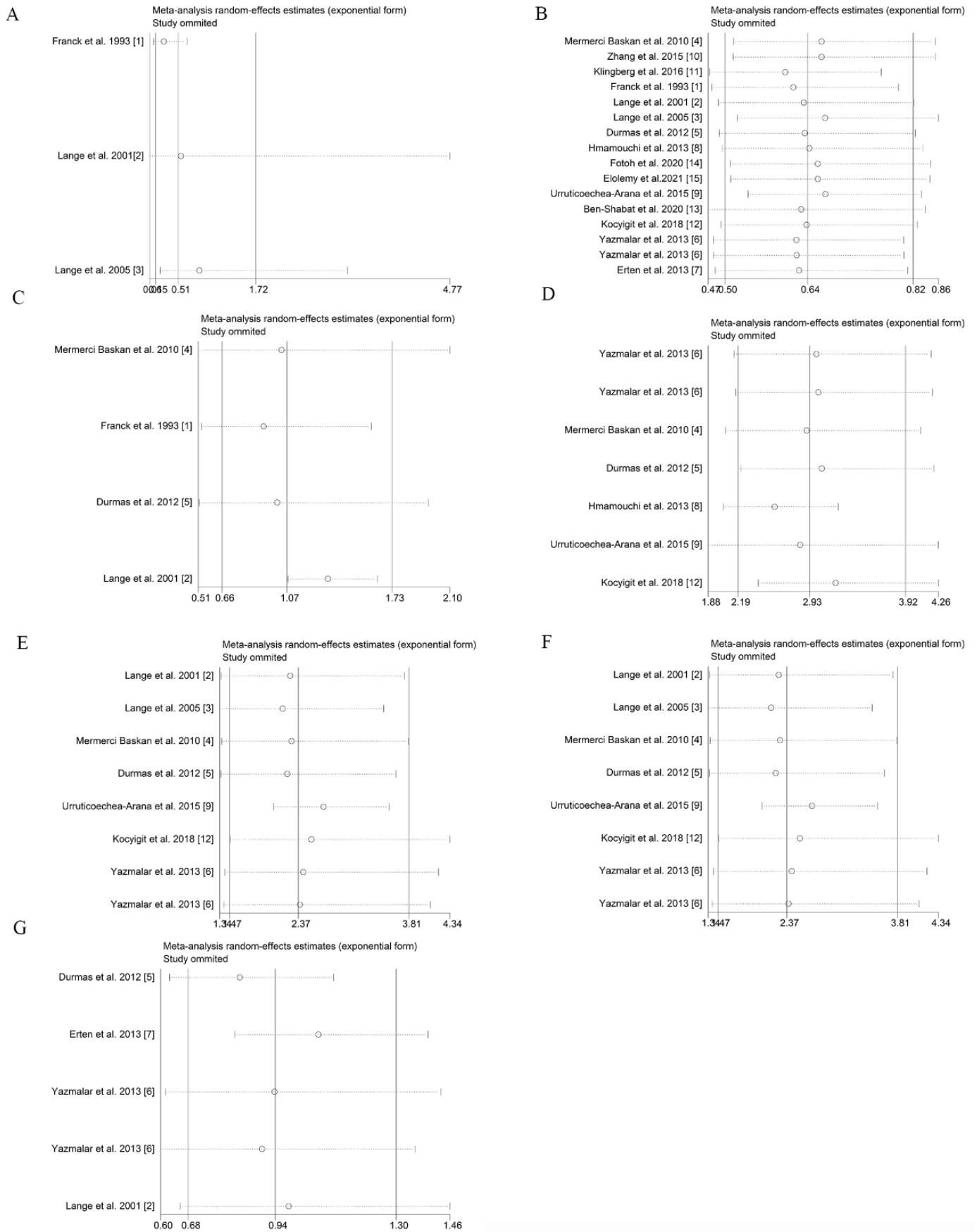

Supplementary figure 3. Sensitivity analyses.

Supplement: Supplementary file 6 [file Image_3.pdf]

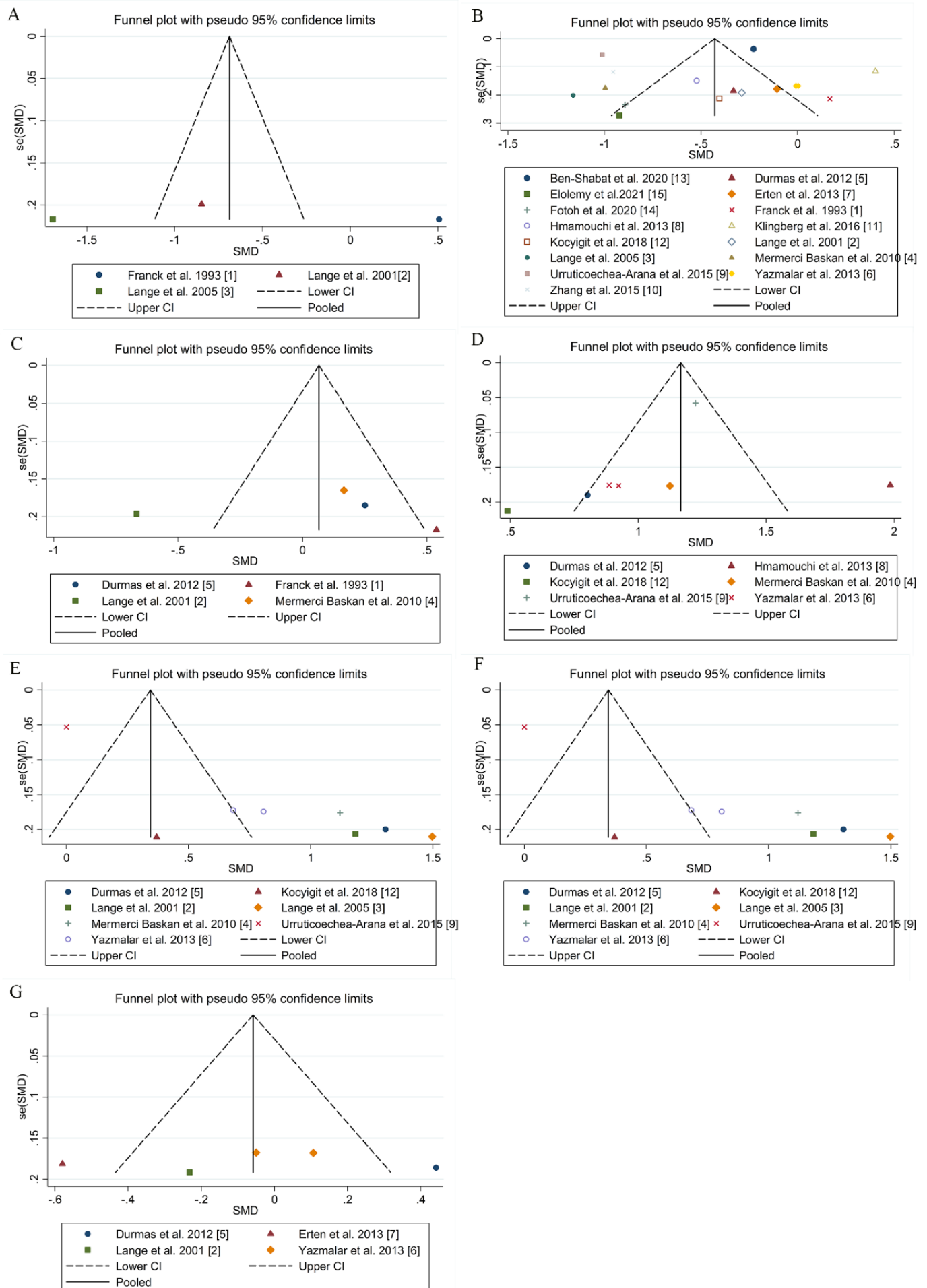

Supplementary figure 4. Funnel plots.

Supplement: Supplementary file 7 [file Image_4.pdf]
