# Supplementary material for: Peripheral vitamin D levels in ankylosing spondylitis: A systematic review and meta-analysis
Source: Front Med (Lausanne). 2022 Aug 26;9:972586. doi: 10.3389/fmed.2022.972586 (PMC9458854; doi:10.3389/fmed.2022.972586)
Supplement: Supplementary file 5 [file Image_2.pdf]

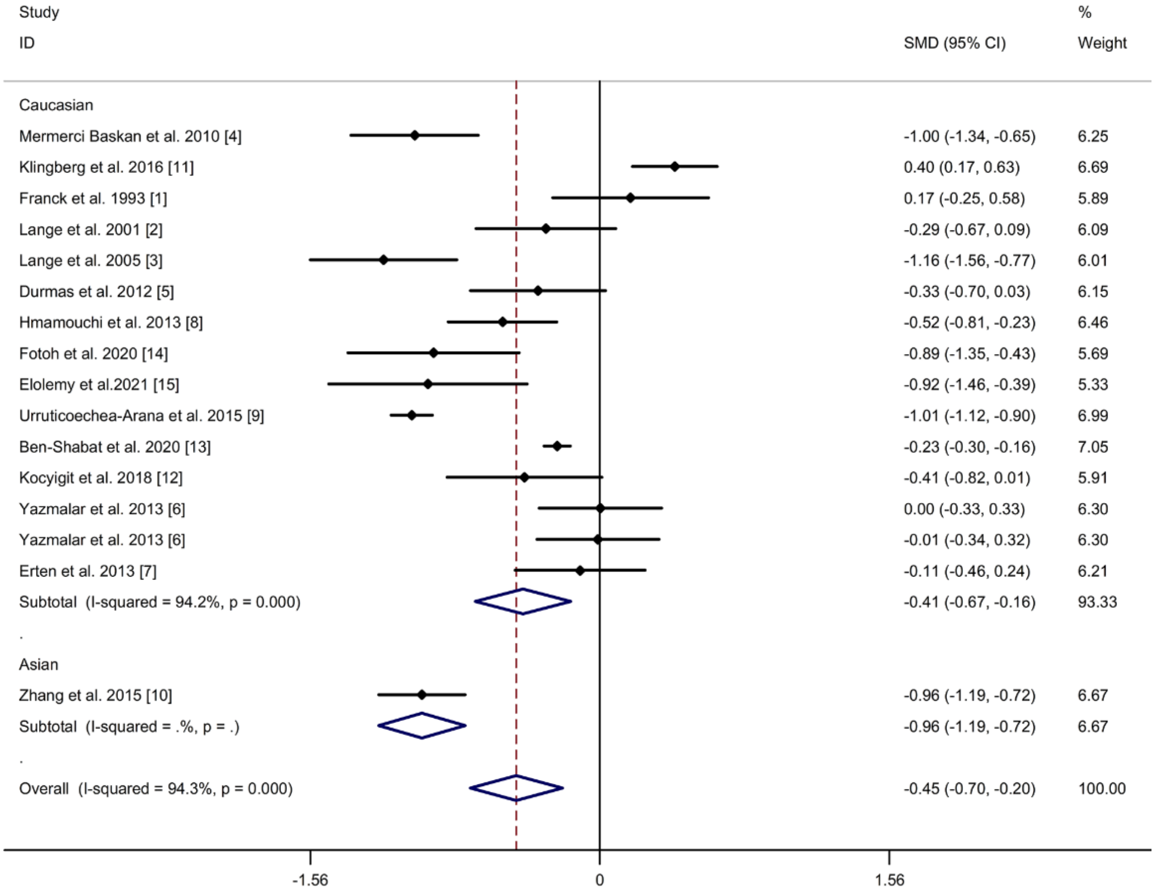

Supplementary figure 2. Subgroup studies comparing peripheral 25OHD levels between AS patients and HC in different ethnicities. Abbreviations: AS, ankylosing spondylitis; 25OHD, 25-hydroxyvitamin D; HC, healthy controls.
